# Genomic Hallmarks of Genes Involved in Chromosomal Translocations in Hematological Cancer

**DOI:** 10.1371/journal.pcbi.1002797

**Published:** 2012-12-06

**Authors:** Mikhail Shugay, Iñigo Ortiz de Mendíbil, José L. Vizmanos, Francisco J. Novo

**Affiliations:** Department of Genetics, University of Navarra, Pamplona, Spain; University of British Columbia, Canada

## Abstract

Reciprocal chromosomal translocations (RCTs) leading to the formation of fusion genes are important drivers of hematological cancers. Although the general requirements for breakage and fusion are fairly well understood, quantitative support for a general mechanism of RCT formation is still lacking. The aim of this paper is to analyze available high-throughput datasets with computational and robust statistical methods, in order to identify genomic hallmarks of translocation partner genes (TPGs). Our results show that fusion genes are generally overexpressed due to increased promoter activity of 5′ TPGs and to more stable 3′-UTR regions of 3′ TPGs. Furthermore, expression profiling of 5′ TPGs and of interaction partners of 3′ TPGs indicates that these features can help to explain tissue specificity of hematological translocations. Analysis of protein domains retained in fusion proteins shows that the co-occurrence of specific domain combinations is non-random and that distinct functional classes of fusion proteins tend to be associated with different components of the gene fusion network. This indicates that the configuration of fusion proteins plays an important role in determining which 5′ and 3′ TPGs will combine in specific fusion genes. It is generally accepted that chromosomal proximity in the nucleus can explain the specific pairing of 5′ and 3′ TPGS and the recurrence of hematological translocations. Using recently available data for chromosomal contact probabilities (Hi-C) we show that TPGs are preferentially located in early replicated regions and occupy distinct clusters in the nucleus. However, our data suggest that, in general, nuclear position of TPGs in hematological cancers explains neither TPG pairing nor clinical frequency. Taken together, our results support a model in which genomic features related to regulation of expression and replication timing determine the set of candidate genes more likely to be translocated in hematological tissues, with functional constraints being responsible for specific gene combinations.

## Author Summary

A common genetic lesion leading to hematological cancer is the creation of fusion genes as a result of reciprocal translocations between chromosomes. Such translocations are non-random, in the sense that certain genes are more likely to be fused than others, and they appear to be tissue-specific. Current models tend to explain the non-random nature of chromosomal translocations suggesting that chromosome breaks are favored at certain sites and that the distance between genes in the nucleus determines the probability of their being fused together. In this work we have analyzed several genomic features in a large collection of genes involved in chromosomal translocations in hematological cancers, using robust computational methods. Our findings suggest that nuclear distance is a general pre-requisite but does not determine the specific combinations of genes fused together. We find that genomic features related to transcription and replication, together with constraints derived from the functional domains present in the proteins encoded by fusion genes, better explain which genes participate in specific chromosomal translocations and the tissue types in which they are found. The association of such genomic features with the position occupied by genes in the nucleus explains the apparent causal role attributed to spatial position.

## Introduction

Chromosomal translocations are genomic rearrangements in which reciprocal exchange of genetic material between two non-homologous chromosomes results in the formation of novel fusion genes. Some of these fusion genes display oncogenic properties and have a strong impact on cancer progression and prognosis, particularly in hematological malignancies [1]. Therefore, several hundred translocations have been described in hematological cancers, although recent reports support their emerging role also in solid tumors [2]. However, whether the fusion gene resulting from a reciprocal chromosomal translocation (RCT) is a driver of tumor progression or just a passenger event is not yet fully understood in all cases [3]. Common and important features of RCTs were reviewed recently [4], but to the best of our knowledge a thorough and extensive data-mining study has not yet been performed.

A fairly comprehensive catalog of genes involved in RCTs is available in public manually-curated databases. Mitelman Database of Chromosome Aberrations and Gene Fusions in Cancer provides extensive documentation of clinical cases, making possible the estimation of clinical frequencies of translocations. TICdb [5], on the other hand, provides manually curated mapped translocation breakpoints, allowing analysis of the sequences flanking those breakpoints. Several such analyses have been performed over the last years, highlighting the association of various sequence motifs with the presence of double-strand breaks (DSB) in some types of translocations. Current consensus about the general requirements for breakage and fusion is that an increased frequency of DSBs at particular genomic locations, together with close spatial proximity of certain loci [6], [7], determine the probability of some RCTs and why some combinations of translocation partner genes (TPGs) are more likely to occur (for a review, see [8]). However, quantitative support for this as a general mechanism applicable to oncogenic RCTs in general is still lacking.

The aim of this paper is to characterize the genomic hallmarks of TPGs using computational and statistical analyses of available clinical and high-throughput datasets. We focus on hematological malignancies, as they constitute the largest set of well-documented RCTs described to date. In this study we have made use of a large collection of data sources. We have used gene expression data for various tissues obtained from publicly available datasets, as well as multiple genomic features from the human genome assembly, to analyze TPG expression and regulation. For the analysis of fusion protein functions we have used InterPro domain features, gene ontology annotations and a recently available protein-protein interaction dataset. We have also used recent high-throughput data on spatial and temporal architecture of the nucleus to search for potential characteristic features of TPGs.

While the mechanisms that could potentially lead to RCT formation are well-studied, it is now emerging that oncogene selection during tumorigenesis could be the decisive factor for RCT appearance in tumors [9]. Thus the logic of this study is to try to explain RCT occurrence from the point of view of functional selection.

First, we compare various features of TPGs with non-translocated genes and find statistical support for some characteristic properties of TPGs related to expression levels and regulation of transcription. We also address the issue of non-random TPG pairing in RCTs, examining issues like nuclear distance, the types of protein domains retained in fusion proteins or replication time. We try to explain some open questions in the field, such as why RCTs are tissue-specific [10] and why they drive oncogenesis in specific lineages [11]. Finally we address the recurrence of RTCs and their clinical frequencies [1]. Taken together, our data, robustly supported by quantitative methods, provide new insights into the global features shared by TPGs and afford a unified explanation of the mechanisms responsible for the specificity and recurrence of RCTs.

## Results

### Expression and regulation of expression of 5′ and 3′ TPGs

**Figure 1:**
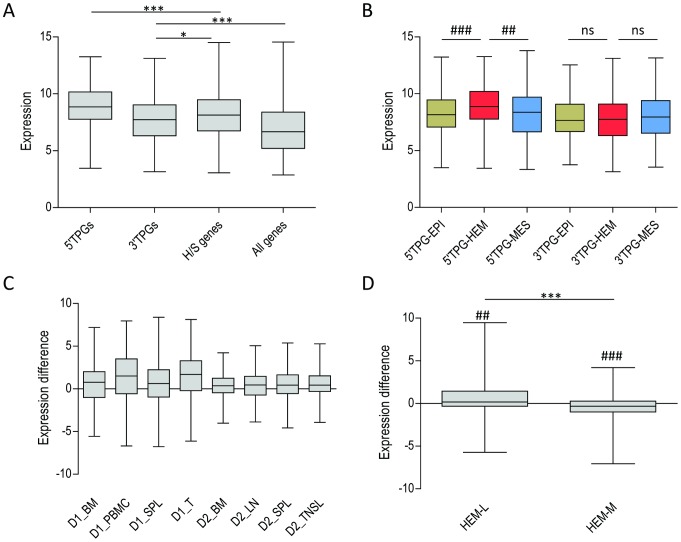
Expression of 5′ and 3′ TPGs. **A**: Average expression in hematological tissues of 5′ and 3′ TPGs compared to expression of all RefSeq genes (all genes) and a subset of genes known to be expressed in hematological tissue (H/S genes, according to UniProt database). **B**: Expression of TPGs in hematopoietic tissues (HEM) compared to non-hematopoietic tissues of epithelial (EPI) and mesenchymal (MES) origin. **C**: Expression difference between 5′ TPGs and 3′ TPGs in various samples of hematopoietic origin from two datasets (D1 and D2, see Methods): bone marrow (BM), peripheral blood mononuclear cells (PBMC), spleen (SPL), T-cells (T), lymph node (LN) and tonsil (TNSL). All differences are highly significant (###). **D**: Difference in expression of 5′TPG between GM128 cell line (of lymphoid origin) and K562 cell line (of myeloid origin) for translocations reported in cells of lymphoid (HEM-L) and myeloid (HEM-M) origin. Higher value means more expression in GM128 compared to K562. Differences are significant in both groups, as indicated by Wilcoxon test. ***, **, *, ns: P<0.001, 0.01, 0.05, Mann-Whitney U test. ###, ##, ns: P<0.001, P<0.01, non-significant, Wilcoxon signed rank test.

Most hematological 5′ and 3′ TPGs are specifically involved in hematological translocations, with only a few of them translocated in other tissue types (10% and 4% for 5′ and 3′ TPGs respectively, according to TICdb). In an attempt to explain this fact, we first analyzed the expression of all TPGs in normal tissues. Expression of both 5′ and 3′ TPGs is significantly higher than average gene expression (all RefSeq genes), but only 5′TPGs (not 3′ TPGs) are more highly expressed than hematopoietic-specific genes in hematological tissues (see Figure 1A and supplementary Figure S1A). Analysis of gene expression in other tissue types revealed that 5′ TPGs, but not 3′ TPGs, are more highly expressed in tissues of hematopoietic origin than in tissues of epithelial or mesenchymal origin (see Figure 1B and Figure S1B). This is true for around 65% of 5′ TPGs. Likewise, when we compared the expression of 5′ TPGs with their corresponding 3′ TPGs we found that 5′ TPGs generally have higher expression levels (see Figure 1C). This was robustly replicated in data from various tissues of hematopoietic lineage and in different microarray datasets for almost 70% of translocations checked. Moreover, we studied lineage-specificity by analyzing expression of 5′ TPGs involved in translocations reported in lymphoid (HEM-L, 177 translocations) and in myeloid (HEM-M, 201 translocations) malignancies. Their expression levels in two cell lines of lymphoid and myeloid origin (GM128 and K562, respectively) confirmed a lineage-specific pattern (only 5′ TPGs in HEM-L show higher expression in GM128 compared to K562), as shown in Figure 1D.

**Figure 2:**
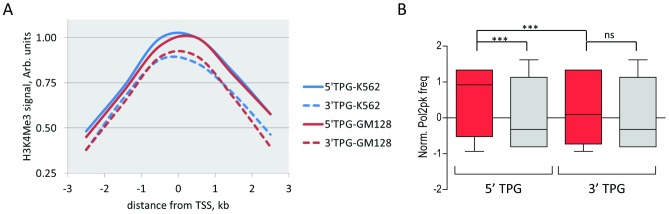
Characteristics of promoters of 5′ and 3′ TPGs. **A**: Averaged histone H3K4 trimethylation mark around promoters of TPGs for K562 and GM128 cell lines (log2 values of signal, normalized to max. value). **B:** Normalized Pol2 peak frequency for 5′ and 3′ TPGs in cell lines of hematopoietic (red bars) and non-hematopoietic (grey bars) origin. ***, ns: P<0.001, non-significant; Wilcoxon signed rank test.

In order to reveal possible mechanisms for such expression differences we analyzed a number of genomic features of TPGs using available tracks from UCSC Genome Browser. Analysis of promoter features in several cell lines suggests that the difference in expression of 5′ and 3′ TPGs could be explained by promoter activity. First, trimethylation of H3K4, which is a marker of active promoters, is higher for 5′ TPGs than for 3′ TPGs in cell lines of hematopoietic origin (Figure 2A). Likewise, we calculated the probability of observing Polymerase II (Pol2) binding in the promoter region of TPGs (−3 kb/+3 kb) as the ratio of cell lines in which a Pol2 peak is found. Normalized Pol2 frequency (ratios were normalized to zero mean and unit standard deviation for all RefSeq genes) was significantly higher in cell lines of hematopoietic origin than in non-hematopoietic for 5′ TPGs, but not for 3′ TPGs. Moreover, Pol2 binding probability was higher in promoters of 5′ TPGs than in 3′ TPGs in hematopoietic cell lines (Figure 2B). These findings suggest that tissue-specific expression of 5′ TPGs could be explained by more active transcription in hematopoietic cells, regardless of other issues such as mRNA stability.

**Figure 3:**
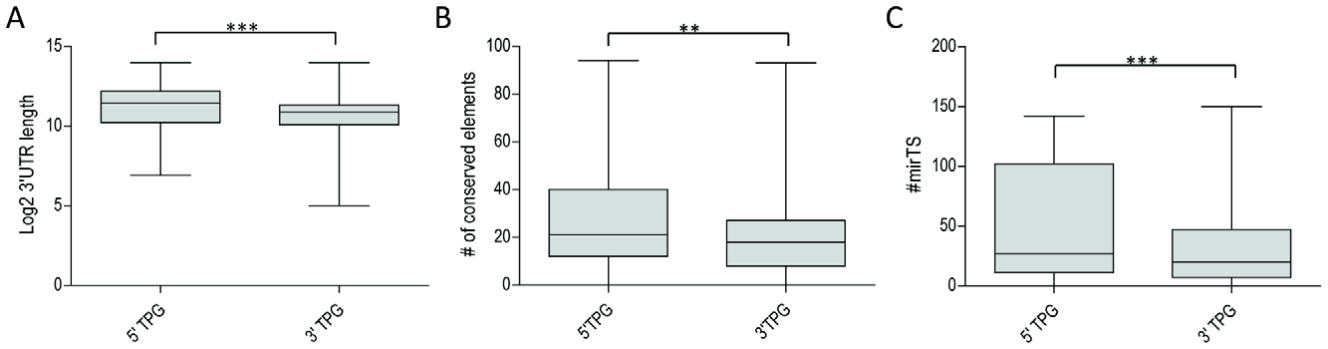
Characteristics of 3′UTRs of 5′ and 3′ TPGs. **A**: 3′UTR length. **B**: number of conserved elements (phastConsElements44way track, hg18 genome assembly from http://genome.ucsc.edu/. **C:** number of microRNA target sites (PITA Top Targets from http://ophid.utoronto.ca/mirDIP. ***, **: P<0.0001, 0.001, Wilcoxon signed rank test.

Since 3′ TPGs contribute their 3′-UTR to fusion transcripts, they could also have an impact on expression levels of fusion genes because 3′-UTRs are known to have a regulatory role in mRNA stability and half-life. For instance, it has been shown that genes more tightly regulated have longer 3′-UTRs with more regulatory elements in them [12]. We explored this by comparing several features of 3′-UTRs of TPGs. We observed that 3′ TPGs have significantly shorter 3′-UTRs than 5′ TPGs. Moreover, there were fewer conserved elements and microRNA target sites in 3′-UTRs of 3′ TPGs (Figure 3). MicroRNAs are known to be major players in post-transcriptional regulation, frequently playing the role of tumor-suppressors by inhibiting expression of proto-oncogenes [13]. All this indicates that 3′ TPGs might also play a role in changing the regulation of expression of oncogenic fusion genes.

### Analysis of domains and protein interaction interfaces

**Figure 4:**
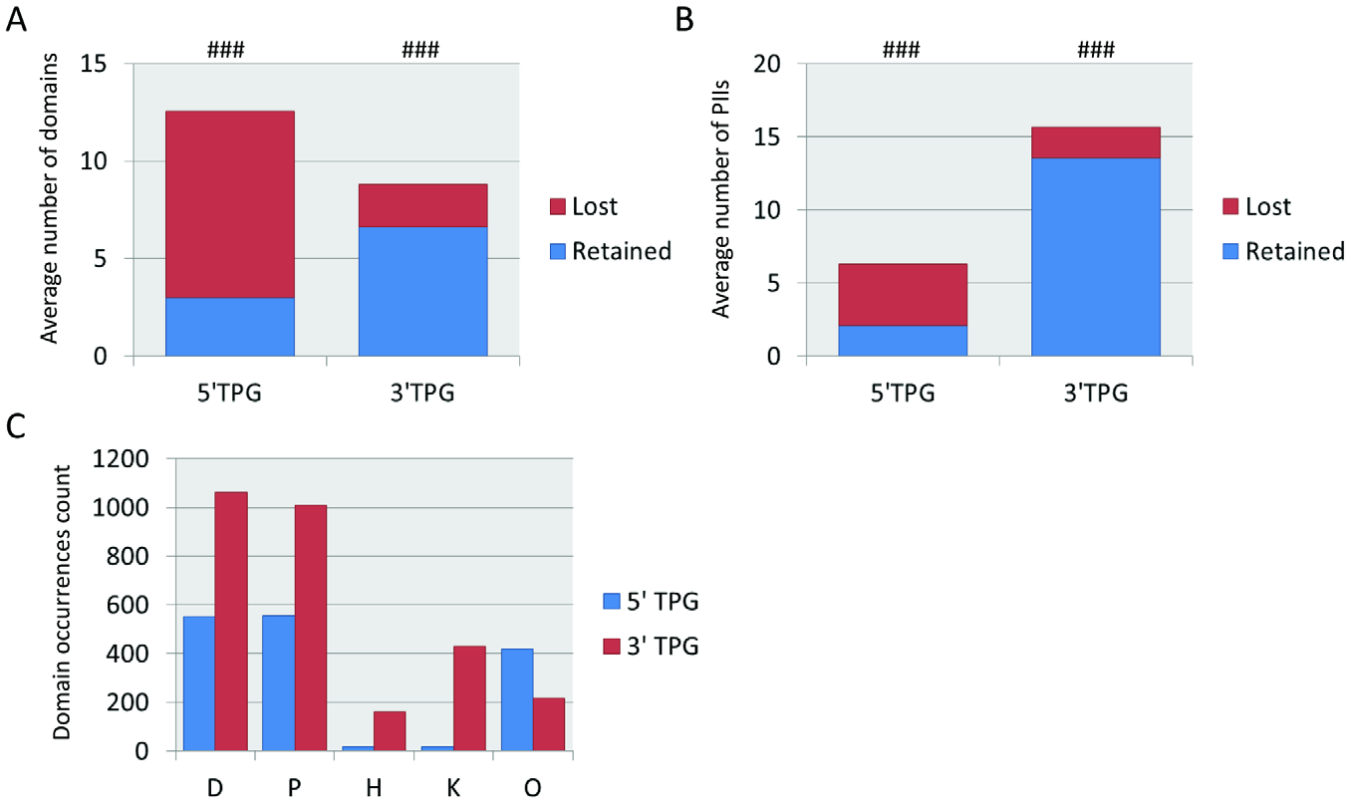
Domains (panel A) and protein interaction interfaces (panel B), retained and lost by fused TPG parts. ###: P<0.0001, Wilcoxon signed rank test for difference between retained and lost elements in a TPG. **C:** Total counts for different types of domains retained by TPGs: DNA-binding (D), protein interaction (P), histone modification (H), kinase (K) or other (O).

We have previously shown that the complex network of TPGs involved in RCTs can be decomposed into a simpler network of protein domain combinations [14], indicating that selection for certain domain combinations is a powerful mechanism driving preferential pairing of TPGs. To further explore this issue, we analyzed the number of domains and protein interaction interfaces (PIIs) that are retained or lost in fusion proteins upon translocation (see Methods for details about extraction of domains and PIIs). While proteins encoded by 3′ TPGs retain significantly more domains and PIIs than they lose, the opposite is true for proteins encoded by 5′ TPGs (Figure 4, panels A and B). In fact, quite a few proteins encoded by 5′ TPGs contained no domains at all, or contained domains that were difficult to associate with oncogenic functions, suggesting that the major role of 5′ TPGs is transcriptional up-regulation of fusion genes rather than contributing specific protein domains to fusion proteins.

**Figure 5:**
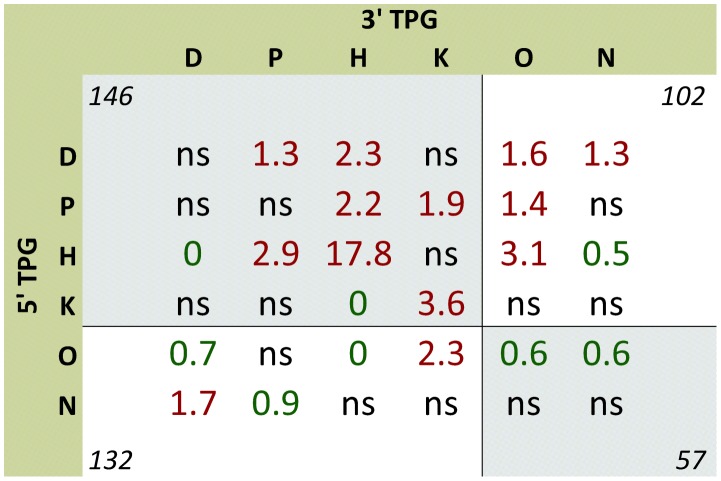
Co-occurrence matrix of various functional classes of domains in hematological translocations. Values in the table show ratio of real count to mean count of permuted data, non-significant ratios are masked as ns. Green and red values represent significantly under- and over-represented co-occurrences, respectively (using 10% FDR cutoff for p-values estimated with 10^6^ random permutations). Values at margins indicate total numbers of translocations with domains of corresponding sets (type 1 domains D, P, H or K and type 2 domains O and N). Types of 5′ TPG and 3′ TPG domain sets are dependent (P = 0.02, two-sided Fisher exact test). Translocations where both TPGs have type 2 domains constitute 13% of all translocations and are under-represented 0.78 fold (P<10^−6^, permutation test).

In order to define this more strictly, we classified protein domains according to their functional ontologies. The details of this manually curated classification procedure are described in Methods and the lists of annotated domains and their classes are given in supplementary Table S1. Type 1 domain classes include DNA-binding (***D***), protein interaction (***P***), kinase (***K***) and histone modification domains (***H***), whereas type 2 domains comprise other types of domains not involved in oncogenesis and domains with unknown function (***O***), as well as protein parts without recognizable domains (***N***). Total numbers of retained domains of each class are presented in Figure 4C, which shows that ***K*** and ***H*** domains are almost absent in 5′ TPGs, whereas ***D*** and ***P*** domains are the most frequent in both 5′ and 3′ TPGs. Likewise, Figure 5 shows that the occurrence of type 1 and 2 domains in 5′ TPGs and 3′ TPGs from the same translocation are strongly dependent, with significant under-representation of type 2-type 2 domain co-occurrences (in agreement with their limited oncogenic potential). Using permutations with a Benjamini-Hochberg FDR cutoff of 10% we discovered 13 over- and 8 under-represented domain class pairs in hematological translocations (Figure 5). Most over-represented pairs of domain classes are in agreement with known mechanisms of oncogenicity of fusion proteins described in the literature (see Discussion).

**Figure 6:**
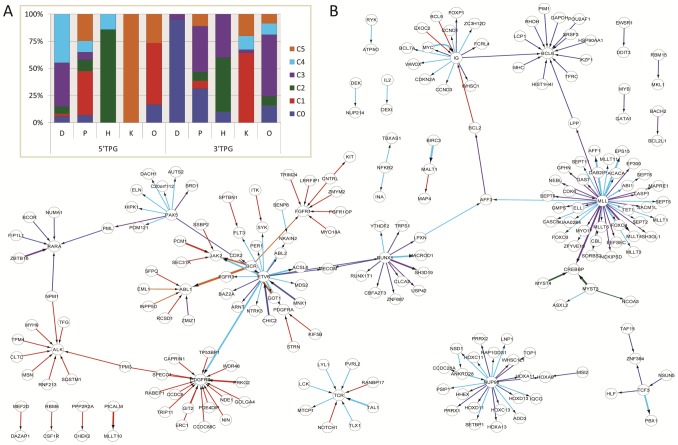
Unsupervised clustering of translocation fusion proteins based on their domain profiles yields 6 translocation classes (C0 to C5). **A**: Relative probabilities of proteins encoded by 5′ or 3′ TPGs to have at least one domain of D/P/H/K/O functional class for each of the six translocation classes. **B**. Network of TPGs (arrows point from 5′ to 3′ TPG) showing how translocation classes are consistent with specific combinations of TPGs (MLL translocations include classes C3 and C4, ALK and PDGFRB translocations are almost exclusively class C1, etc). Edge thickness indicates number of different TPG variants comprising a given translocation.

To further explore the non-randomness of domain co-occurrence in translocations for more complex sets than domain pairs, we performed clusterization of fusion protein functional profiles, defined as binary strings where each bit indicates whether a TPG retained at least one domain of a certain class. Clusterization using an expectation-maximization algorithm with cross-validation yielded an estimate of n = 6 clusters, with four clusters (C0, C2, C3 and C4) containing fusions with “regulation of transcription” signature and the other two (C1 and C5) having a “kinase” signature. Notably, small clusters C2 and C5 include only true chimeric proteins in which both TPGs have similar functions. Figure 6A shows the resulting cluster profiles and counts. We then overlaid these clusters onto the network of fusion genes (Figure 6B; for each translocation, edge color indicates the cluster to which it belongs). This shows that clusters tend to be restricted to non-overlapping sets of hubs and that events where the same gene belongs to translocations of both “regulation of transcription” and “kinase” clusters are extremely rare.

**Figure 7:**
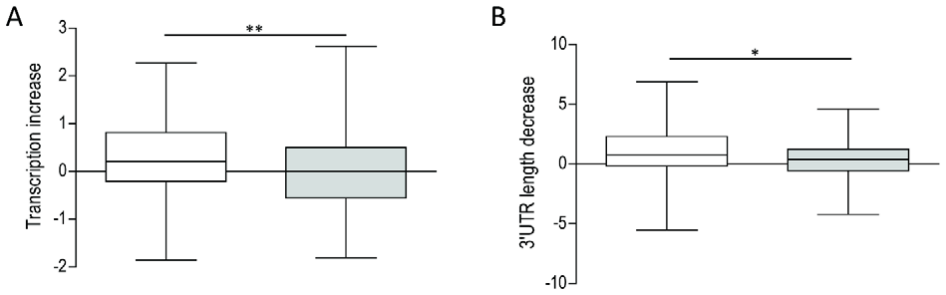
TPGs with type 2 domains (not known to be involved in DNA-binding, protein-interaction, kinase or histone modification) play a role in boosting transcription and mRNA stability of fusion gene. Comparison of TPGs with type 1 domains (grey boxes) and type 2 domains (white boxes). **A:** Transcription increase afforded by 5′ TPGs, calculated as ((Pol2 occupancy of 5′TPG promoter)−(Pol2 occupancy of 3′TPG promoter)). **B:** Decrease of 3′-UTR length afforded by 3′ TPGs, calculated as log2((3′-UTR length of 5′ TPG)/(3′-UTR length of 3′ TPG)). *, **: P<0.05, 0.01; two-tailed T-test.

To investigate the role of TPGs containing only type 2 domains we analyzed their predicted impact on the expression of their corresponding fusion genes. As shown above, 5′ TPGS are characterized by higher expression levels whereas 3′ TPGs are characterized by shorter 3′-UTRs. Thus, we computed the relative difference of expression between 5′ TPGs and 3′ TPGs from different translocations based on the presence of type 1 or type 2 domains in 5′ TPGs. Similarly, we calculated the decrease in length of the 3′-UTR of fusion proteins in translocations with 3′ TPGs containing or lacking type 1 domains. In both cases, we found evidence (Figure 7 and Figure S2) that TPGs without type 1 domains can lead to overexpression of the fusion gene (in the case of 5′ TPGS) or improved stability of its mRNA (for 3′ TPGs).

**Figure 8:**
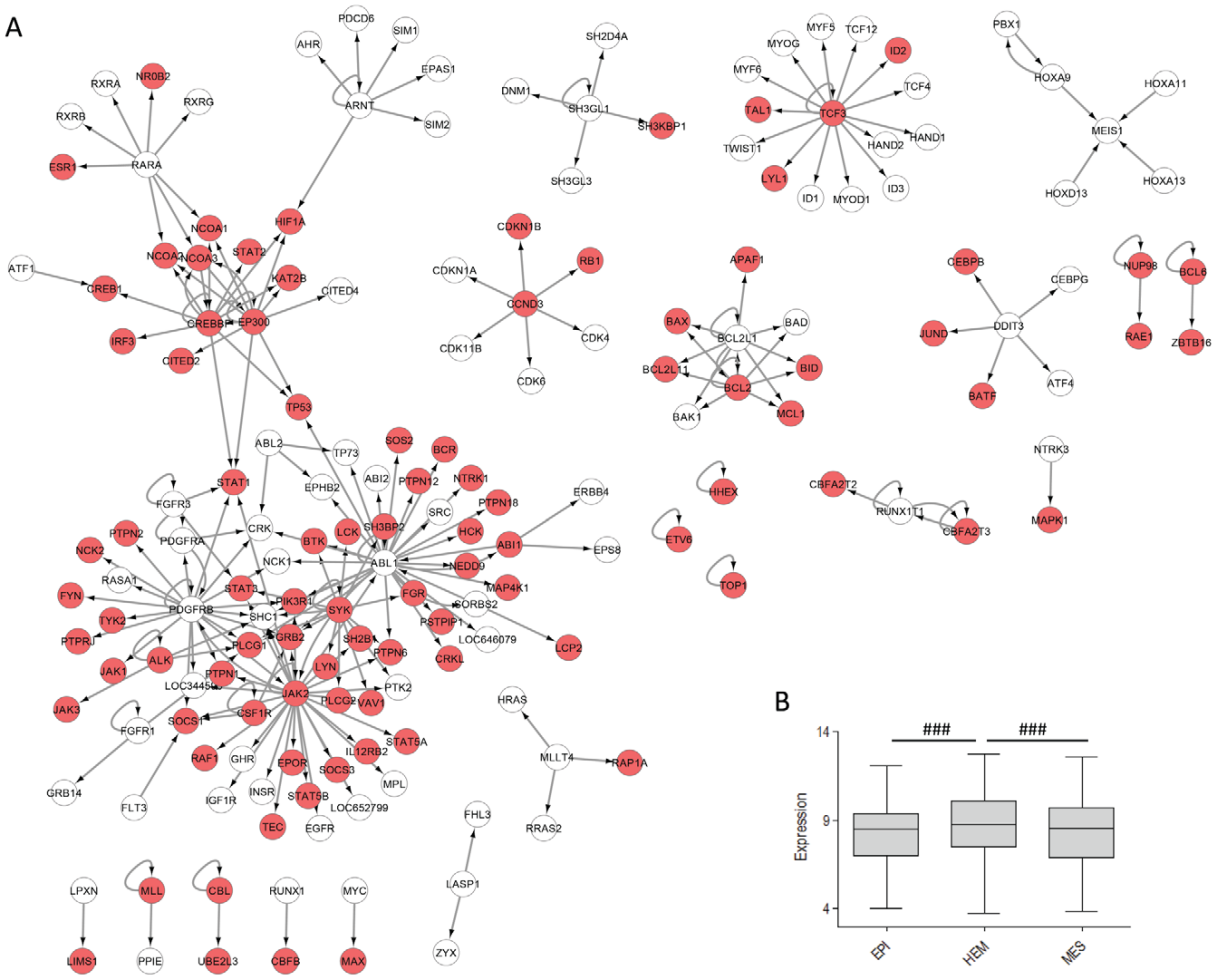
Analysis of interaction partners of 3′ TPGs. **A**: Network of protein interaction interfaces (PIIs) retained by 3′ TPGs and their interaction partners. Edges point from a 3′ TPG towards its interacting proteins. Red nodes indicate genes that are on average more expressed in hematopoietic than in non-hematopoietic samples. **B**: Expression of interacting partners in tissues of epithelial (EPI), hematopoietic (HEM) and mesenchymal (MES) lineage. If multiple 3′TPGs have the same interacting protein, it is included the corresponding number of times. ### - p<0.001, Wilcoxon signed rank test.

As mentioned above, expression levels of 3′ TPGs could not explain why most of them are translocated exclusively in hematological tissues. To explore other possible ways in which 3′ TPGs could contribute to tissue-specificity, we identified all protein interaction interfaces (PII) in proteins encoded by 3′ TPGs, and extracted data about the proteins that interact with those PIIs. With this information we built a network of interactions between PIIs in 3′ TPGs and their interaction partners, shown in Figure 8A (the network for 5′ TPGs is shown in supplementary Figure S3). This network has a complex structure with many self-interactions and overlaps between interaction partners of different TPGs, many of which comprise large hubs. We then calculated the expression of these interaction partners in several tissues in the same expression datasets that we had used before. Interestingly, expression levels of interaction partners of 3′ TPGs were, on average, significantly higher in hematopoietic than in epithelial or mesenchymal tissues (Figure 8B and supplementary Figure S4). Likewise, 84% of 3′ TPG interaction partners are highly expressed in hematopoietic stem cells from bone marrow and 70% to 80% of them are highly expressed in various peripheral blood cells, according to annotations in DAVID database [15] (GNF_U133A_QUARTILE used, these percentages are significant with Benjamini-corrected P-values less than 0.05). Taken together, these results suggest that 3′ TPGs might influence tissue-specificity of translocations indirectly, via tissue-specific expression of the proteins interacting with them.

### Spatial position in nucleus

**Figure 9:**
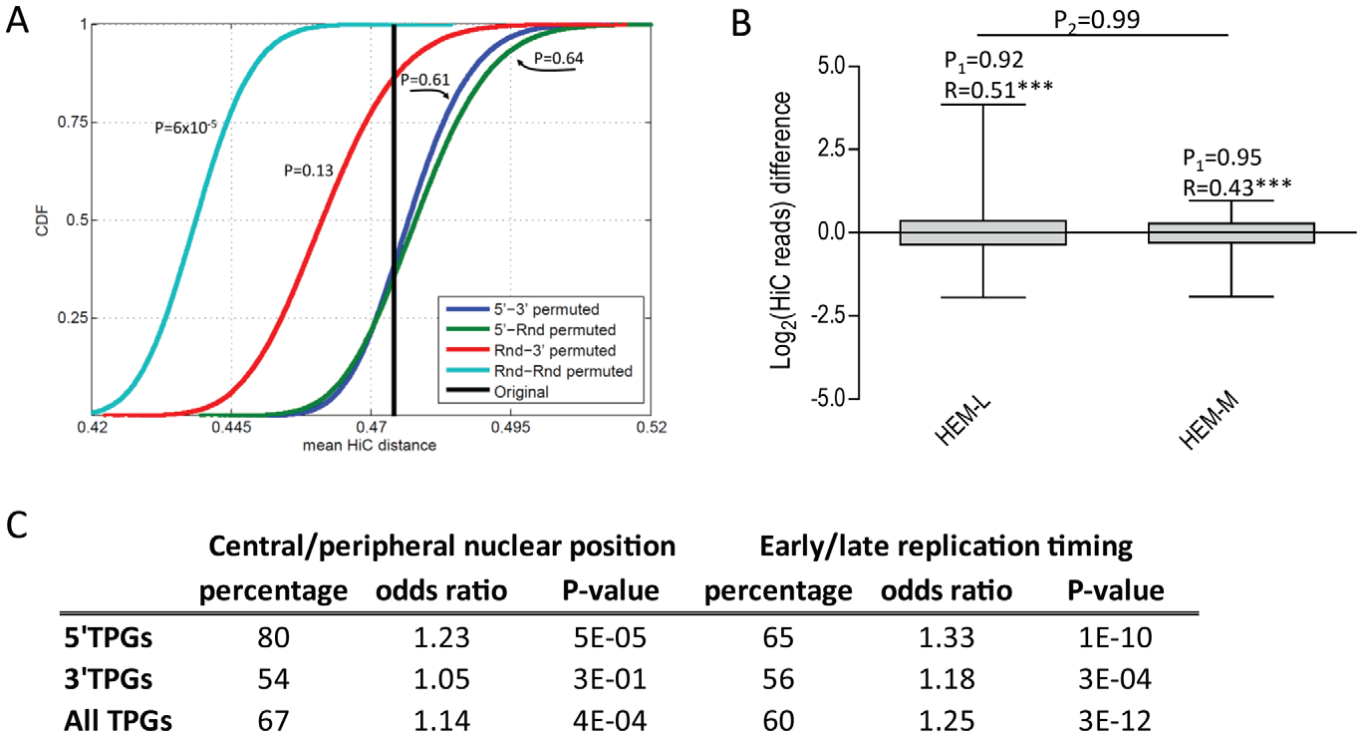
Spatial positioning and replication timing of TPGs. **A:** Mean pairwise Hi-C distance distributions (for n = 239 TPG pairs) obtained using various permutation schemes (N = 10^5^ permutations); black line: original TPG pairs; blue: 5′ and 3′ TPGs randomly shuffled; red and dark green: either 5′ or 3′ TPGs replaced by random Refseq genes (Rnd); light green: random Refseq genes of the same size chosen arbitrarily as 5′ or 3′ TPG. Computed P-values for mean Hi-C distance of the original TPG set against permuted distributions are shown in the plot. **B:** Spatial closeness of TPG loci from translocations reported in tumors of lymphoid (HEM-L) and myeloid (HEM-M) origin, computed based on Hi-C data for GM06990 (of lymphoid origin) and K562 (of myeloid origin) cell lines. Difference between log2(Hi-C reads) of GM06990 value and K562 value is shown (higher value means closeness in GM06990 compared to K562). Wilcoxon matched ranks test P-values (P_1_) and Spearman correlation coefficients (R) computed based on pairwise comparisons of distances for each translocation in HEM-L and HEM-M set. Correlation of Hi-C data between two cell lines is highly significant (***, P<0.001, T-test). Hi-C closeness differences are statistically the same for HEM-L and HEM-M (P_2_, Mann-Whitney test). **C:** TPG frequency in central (proximal) and early replicating genomic loci, based on Hi-C and RT data respectively. Percentage of TPGs in central (early) genomic loci and odds ratios of these percentage compared to background set (all RefSeq genes) are presented. P-values computed based on binomial distribution.

Preferential fusion of certain 5′ and 3′ TPGs in hematological cancers is generally attributed to the spatial proximity of those loci within the nuclear space. This has been demonstrated for a few recurrent translocations [8], but the relevance of spatial proximity as a general feature capable of driving specific translocations in hematological malignancies has not been assessed on a large scale. We therefore used high-throughput spatial proximity data (represented as interchromosomal contact frequencies) available for human lymphoblastoid cell line GM06990 [16], [17], in order to check whether known 5′-3′ TPG pairs are closer in the nucleus, on average, than random genes. As shown in Figure 9A, mean contact frequency calculated for 239 TPG pairs (black line) is significantly higher than for randomly selected Refseq genes (light green), indicating a preferential position in a contact-enriched zone of the nucleus. However, neither replacing a 5′ or a 3′ TPG with a random Refseq gene (red and dark green lines) nor, more importantly, randomly permuting 5′-3′ TPG pairs (blue line), significantly decreased the average distance between paired genes. This suggests that spatial proximity, in itself, does not have a major role in determining the preferential pairing of specific TPGs in hematological neoplasms.

A more refined way to test the relevance of spatial proximity for lineage-specificity of translocations is to select translocations reported only in lymphoid or in myeloid malignancies (HEM-L and HEM-M subsets mentioned above) and to measure the distance between those loci in cell lines of lymphoid and myeloid lineage. Therefore, we compared distances between TPG pairs in GM06990 cells (lymphoblastoid origin) and in K562 cells (myeloid origin), for which Hi-C data were also available. As shown in Figure 9B, we found no lineage-specific trends: TPG pairs from HEM-L and HEM-M translocations were at similar distances in GM06990 and K562 cells. Moreover, we observed very significant correlation of distances between cell lines (see Figure 9B).

To gain further insights into the relative positions of TPGs in the nucleus, we performed clusterization of Hi-C distances to obtain clusters corresponding to central and peripheral locations (supplementary Figure S5). We found that 5′ TPGs are significantly over-represented in the central (proximal) cluster of the nucleus (Figure 9C, supplementary Figure S5C). Likewise, 5′ TPGs are, on average, closer to all other nuclear loci (excluding loci on the same chromosome) than RefSeq genes, according to their higher mean contact frequency (supplementary Figure S5E). This, in contrast, was not observed for 3′ TPGs, indicating that 5′ TPGs occupy a more central position in the nuclear space whereas 3′ TPGs are more evenly distributed. The overall association of TPGs with regions in the central cluster is shown in supplementary Figure S5D.

Replication timing (RT) is highly correlated, at the chromosomal level, with the organization of chromosomal domains within territories [18]. Furthermore, the RT of a chromosomal region is associated with mutation rate [19] and somatic copy number alterations are often bounded by early replicating regions [20]. We grouped smoothed RT data for GM06990 cell line into early and late replicating regions based on distribution of all RefSeq genes (supplementary Figure S6) and analyzed RT values of TPG-containing regions. As shown in Figure 9C and supplementary Figure S6B, both 5′ TPGs and 3′ TPGs are significantly over-represented in early-replicating regions. Likewise, TPGs are replicated significantly earlier than all RefSeq genes based on raw RT values for their genomic regions (supplementary Figure S6D), with 5′ and 3′ TPGs having similar timing. To further strengthen this observation, we repeated our analysis on more samples from the ReplicationDomain database. The results obtained for hematological samples were in good agreement with GM06990 cell line, while non-hematological samples only show slight trends (supplementary Figure S6C).

### Translocation features related to clinical frequency

**Figure 10:**
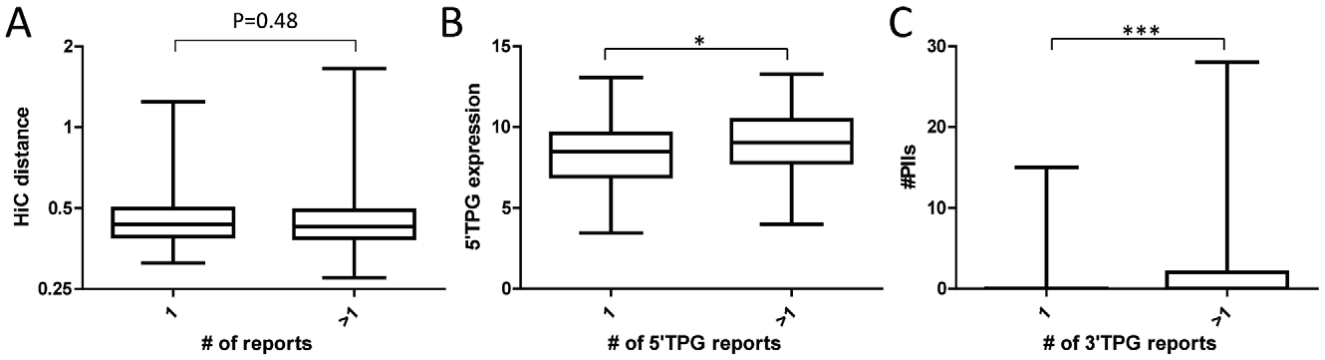
Clinical frequency of chromosomal translocations is influenced by some features of TPGs but is not dependent on their spatial proximity. **A**. Hi-C distance (calculated as contact frequency) between TPG-containing loci for rare (reported once) and frequent (reported more than once) translocations. **B**: expression levels of rare and frequent 5′ TPGs. **C**: number of protein interaction interfaces in rare and frequent 3′ TPGs. *, ***: P<0.05, 0.0001; All P-values calculated using Mann-Whitney U test.

Another relevant question about the forces that drive specific combinations of TPGs in hematological malignancies is why some translocations are more frequently found in clinical samples whereas other translocations are never, or rarely, recurrent. It has been previously argued [7] that clinical frequency (estimated from the number of reported cases in Mitelman database) is correlated with spatial proximity for rearranged loci, leading the authors to suggest that high-order spatial genome organization, with non-random localization of these loci in relation to the center of nucleus, might increase their spatial proximity. Therefore, we extracted data for all translocations reported in hematological malignancies in Mitelman database and computed the contact frequency of various combinations of genes. First, we found that the average distance between TPGs in rare translocations (reported only once) is the same as the distance between TPGs in frequent translocations (reported more than once) (Figure 10A). Furthermore, when we compared RT and contact frequency across the whole genome (excluding their own chromosome) for TPGs in rare and frequent translocations we did not observe any significant difference, as shown in supplementary Figure S7 (which shows separately 5′ TPGs and 3′ TPGs). Again, this observation suggests that spatial proximity is not sufficient to account for the recurrence of specific translocations, and that additional factors must be considered.

We therefore evaluated the role that other TPG properties might play in increasing the frequency of certain translocations. First, we found that expression levels of 5′ TPGs were significantly higher in recurrent translocations (reported more than once) than in rare (Figure 10B). Similarly, we explored whether the number of known PIIs in 3′ TPGs might be associated with the frequency of their involvement in translocations. We found that 3′ TPGs reported more than once have significantly more PIIs than rare 3′ TPGs (Figure 10C).

## Discussion

### TPG features that drive oncogenic translocations

Chromosomal translocations frequently lead to cancer development via promoter substitution and loss of transcriptional control [21]. In agreement with this, we find that TPGs display significantly increased expression levels. In the case of 5′ TPGs, this seems to be the result of increased promoter activity, as shown by the presence of significantly more H3K4me3 marks and pol2 peaks near the TSS and evidence early replication timing. As regards 3′ TPGs, we found that they have shorter 3′-UTRs with less regulatory elements than corresponding 5′ TPGs, thus facilitating the escape of fusion genes from post-transcriptional control. In this regard, it has been previously reported that some chromosomal translocations lead to oncogenic transformation by disrupting microRNA-mediated gene repression [22] and that replacing the 3′-UTR of *MLL* with 3′-UTRs of its TPGs removes post-transcriptional inhibition of its expression [23]. Such loss of post-transcriptional control could contribute to the oncogenicity of *MLL* translocations. We propose that a similar mechanism could be important, more generally, to determine the oncogenic potential of translocations in hematological neoplasms. This confirms that genes involved in fusions are generally selected because they cause overexpression of oncogenes, as seen for many mutations [4], [21].

It has also been shown that co-transcriptional processes can influence genome stability due to transcription-induced R-loops in which activation-induced-deaminases (AID) introduce DNA breaks [24]. Indeed, such a mechanism has been invoked to explain the presence of RCTs in B-cell malignancies [25], [26]. More recently, analysis of the genomes of 21 breast cancer samples has revealed that regions of somatic hypermutation tend to associate with breakpoints of somatic rearrangements [27], again suggesting that AID enzymes might play a role in co-transcriptional generation of DNA breaks. Thus, transcription might establish a link between gene involvement in RCTs and DSB frequency across the genome. This, however, would not explain why 3′ TPGs with moderate expression levels are preferred over other hematological tissue-specific genes. This could be explained by other mechanisms leading to the generation of DNA breaks which are not directly related to high transcription rates, such as replication. It is now clear that DNA breaks leading to some forms of chromosomal rearrangement can be induced during replication [28]. However, recent studies in yeast show that error-prone DNA due to template switching is associated with late-replicating regions [29], while we observe that TPGs are over-represented in early-replicating regions. Given that early-replicating regions contain active genes with important cellular functions [30], this is reminiscent of the master gene hypothesis according to which genes involved in RCTs are chosen because of their functional attributes [31].

In this regard, we have previously suggested that the need to combine certain protein domains imposes selective constraints on fusion proteins, so that those combinations with greater oncogenic potential are more frequently found in tumor cells [14]. Similarly, other authors have found that features such as intrinsic structural disorder of fusion proteins are important for their oncogenic properties [32]. However, the role played by functional selection is usually overlooked in models that try to explain specific combinations of TPGs in oncogenic translocations. Here, we have found that oncogenic translocations retain a significant percentage of domains and protein interaction interfaces of 3′ TPGs. We further used InterPro annotations to classify all protein domains into five broad classes, assuming that an oncogenic fusion protein should contain at least one domain with oncogenic properties. Such classification scheme is supported by the fact that fusion proteins in which both TPGs contain “type 2” domains (that is, domains not belonging to any of the four major functional categories with oncogenic potential) are significantly under-represented. Among over-represented domain combinations are the co-ocurrence of protein interaction domains (many of which are capable of protein oligomerization) with DNA-binding and kinase domains, as well as co-occurence of two kinase domains, which result in aberrant cellular signaling [33]. Likewise, our data show that co-occurrence of histone modification and protein interaction domains is non-random, which is consistent with the important role played by aberrant chromatin modification in cancer [34], and in particular in translocations involving *MLL* [35], [36].

Interestingly, most over-represented domain co-occurrences involve one type 1 and one type 2 domain, indicating that in most fusion genes one of the TPGs is simply promoting overexpression of the fusion protein. A 5′-TPG with type 2 domains could contribute a strong promoter, whereas a 3′-TPG with a type 2 domain might stabilize the fusion mRNA by contributing its 3′-UTR. Our analysis of expression gain of 5′ TPGs, as well as potential mRNA stability gain of 3′ TPGs, suggests that both mechanisms are generally operative.

Finally, to obtain a global view of the relative contributions of the various domain features on non-random fusion partner selection, we performed an unsupervised classification of fusion proteins based on the functional signatures of their 5′ and 3′ TPGs. The superimposition of these functional clusters onto the fusion network reveals that functional profiles of fusion proteins robustly capture the basic structure of the network.

Our data also suggest that tissue-specificity of translocations might be explained, at least in part, by tissue-specific expression of TPGs. First, we found that 5′ TPGs involved exclusively in hematological neoplasms are significantly more expressed in hematopoietic tissue compared to epithelial and mesenchymal tissues, which is somehow expected because the oncogenic potential of fusion genes relies on their expression levels in the tissue where the translocation takes place. But when we analyzed 5′ TPGs involved in translocations reported only in lymphoid or in myeloid neoplasms, comparing their expression levels in two cell lines of lymphoid and myeloid origin (GM128 and K562, respectively), our results confirmed that expression of 5′ TPGs is an important contributor to tissue specificity of RCTs. As for 3′ TPGs, we found that their contribution to tissue specificity is dependent on the expression levels of their interaction partners, which are significantly higher in hematopoietic than in non-hematopoietic tissues according to all gene expression datasets analyzed.

### Spatial proximity of TPGs in the formation of specific chromosomal translocations

It is interesting that some of the genomic features of TPGs that we have found correlated with tissue-specificity of translocations are known to be related to the spatial organization of the genome. Transcriptional activity, for instance, clearly affects the position of genes: inactive genes inside a chromosome territory are constrained in their mobility and thus in their potential to interact with distant loci [37], so that the ability of actively transcribed regions to interact *in trans* requires that those regions loop outside of their respective territories [38]. Likewise, replication timing is highly correlated with nuclear position and chromosome architecture [39]–[41]. It is well documented that chromosomes occupy spatially defined territories in the nucleus [42] so that intra-chromosomal contacts are more frequent than inter-chromosomal [16]. This organization is tissue specific with some intra-chromosomal contacts being more frequent in some tissues [6], [43]. Thus it has been speculated that the recurrence and tissue-specificity of translocations could be explained by the spatial proximity and physical contact frequency of translocated loci [6], [7]. Our observation that 5′ TPGs are preferentially found in transcriptionally active domains in the center of the nucleus could provide a link between high-order genome organization and the potential occurrence of translocations, because a central position within the nuclear space (which is known to be tightly linked with transcriptional activity) increases average contact frequency with other loci [44], [45].

However, our results suggest that spatial proximity *per se* is not a decisive factor in determining specific combinations of TPGs and their clinical recurrence. Although it has long been speculated that spatial proximity determines the specific pairing of TPGs in translocations [8], data supporting this contention as a general mechanisms for all types of translocations is lacking. For instance, Parada et al. [6] used mouse chr5:chr6 and chr12:chr15 chromosome pairs to show that the frequency of translocations is correlated with the frequency of chromosomal contacts. These chromosome pairs provide a useful model, as chromosomes 5 and 6 are known to be translocated in hepatocytes but not in lymphocytes, while the opposite is true for chromosomes 12 and 15. These authors showed that contacts between chr5:chr6 are significantly more frequent in hepatocytes, while chr12:chr15 contacts are significantly more frequent in lymphocytes. But recent Hi-C data provides a more global and accurate measure of contact frequencies between chromosomal loci in a human lymphoblastoid cell line [16]. Using the distribution of interchromosomal contact frequencies for all pairs of loci between two chromosomes as a measure of their closeness, we found that chr12:chr15 (which are known to be translocated in human hematological malignancies) are closer than chr5:chr6 (not reported to be translocated). However, we also observed that chromosomes 5 and 2, frequently rearranged in translocations involving the *ALK* gene in anaplastic large cell lymphoma cases [46], were even farther apart in the nucleus than chromosomes 5 and 6 (supplementary Figure S8A). When this analysis was extended to all translocations and all loci, we found no difference between the average distances for chromosome pairs that are rearranged in hematological malignancies versus non-rearranged pairs (supplementary Figure S8B). Thus, while TPGs are non-randomly distributed relative to the center of the nucleus, we propose that their pairing in specific combinations is mainly driven by other factors such as gene activity, which (through their association with high-order genomic organization) lead indirectly to their nuclear closeness (supplementary Figure S8C).

In this view, spatial proximity is a necessary pre-requisite for the appearance of a translocation, but it is unlikely to be the only (or even the most important) factor to explain the specificity and recurrence of oncogenic translocations [47]. In fact, our data show that the specificity of TPG pairing and the recurrence of specific gene pairs are not directly dependent on spatial proximity. Recent findings in the budding yeast have shown that broken chromosomal ends created by DSBs are able to travel relatively long distances within the nuclear space to search for homologous templates. Miné-Hattab and Rothstein [48] have demonstrated that after induction of a DSB the broken chromosome explores up to 30% of the nuclear volume in diploid cells, about 10-fold larger than the volume to which chromosomes are constrained in the absence of breaks. A similar observation was made by Dion et al [49] in haploid cells, where DSBs stayed for hours searching for a homologous template. These results show that it is possible to join regions which are relatively distant in the nucleus, and are consistent with our contention that spatial proximity is not a strong determinant of translocation frequency or specificity in hematological neoplasms on a global scale. Therefore, the specificity of TPG pairing could be better explained by the preferential positioning of 5′ TPGs in the central zone of the nucleus, which in turn is related to functional features such as gene expression and replication timing. This central location might put these genes within the nuclear distance required to undergo a translocation with several potential 3′ TPGs. Then, selection for fusion proteins with oncogenic potential will dictate which specific gene pairs are eventually found in a particular tissue, and their relative frequency in patient samples.

The issues discussed here also have important practical implications. Several studies published over the last few years have demonstrated the ability of next generation sequencing (NGS) to identify novel fusion transcripts in cell lines and in samples from patients with hematological and solid cancers [50]–[52]. However, the functional significance of newly identified fusions is not always clear because it is possible that many of these novel RCTs are the result of an increased background of genomic instability, rather than being driver oncogenic events [47], [51], [53]. Thus, there is a need for methods that identify which of these tumor specific RCTs are required for establishment and maintenance of the transformed phenotype [52]. The genomic features of 5′ and 3′ TPGs that we have identified in this work might help to develop computational approaches for the prediction of fusion genes that are more likely to have a causal role in the initiation or progression of hematological neoplasms.

## Methods

### Selection of translocations and partner genes

Data from translocations, including the Ensembl transcript ID of translocation partner genes and the nucleotide position of breakpoints, were extracted from TICdb v3.1 (http://www.unav.es/genetica/TICdb/). There are some translocations involving the same gene pair with different breakpoint positions, which is redundant for some types of analysis. Therefore in some cases we have used unique TPG instances for expression studies, and unique TPG pairs for analysis of genome organization features. For analysis of the functions of fusion proteins we have used TPG pairs that were unique in terms of InterPro domain composition. We successfully extracted from TICdb data for 770 hematopoietic translocation entries involving 1175 TPGs, comprising 245 TPG pairs with unique gene names (117 unique 5′TPG and 161 unique 3′TPGs). Translocations and TPGs for which certain data were not available (e.g. expression or genomic organization) were not included in the corresponding analysis. For lineage-specific analysis, translocations reported in malignancies of lymphoid (HEM-L, 177 translocations) and myeloid (HEM-M, 201 translocation) origin were selected.

### Expression data analysis

Human gene expression data (dataset #1) for three tissue lineages, epithelial (EPI), hematopoietic (HEM), or mesenchymal (MES), was extracted from Gene Expression Omnibus (GEO, www.ncbi.nlm.nih.gov/geo/) as follows. For each tissue lineage we manually selected four tissues as samples: colon mucosa (GSE8671), lung epithelium (GSE30660), mammary epithelium (GSE25487) and pancreatic duct epithelium (GSE19650) for EPI; bone marrow (GSE32057), peripheral blood mononuclear cells (GSE11281), spleen (GSE25550) and CD3+ T-cells (GSE6088) for HEM; omental adipose tissue (GSE3526), meniscal cartilage (GSE19060), skin fibroblasts (GSE20538) and aortic vascular smooth muscle cells (GSE11367) for MES. Gene expression levels for each of four tissue samples were calculated as the average of up to three randomly selected donor samples available in microarray data, using only data for non-malignant tissues. All microarray data were first downloaded as .CEL files and then jointly normalized by RMA express (http://rmaexpress.bmbolstad.com/). Analysis was replicated in an independent dataset (dataset #2) using human gene expression atlas [54] as an alternative source of data. For that purpose the whole dataset was downloaded as a normalized matrix file from GEO and then manually grouped by tissue type. Overall microarray data from both the manual dataset and gene atlas were in good agreement and yielded similar results. Thus we present here only graphs generated using dataset #1, providing graphs for dataset #2 as supplementary information. For analysis of lineage-specific expression patterns of 5′TPGs we used expression data in GM12878 and K562 cell lines from ENCODE project (GSE26312).

### Analysis of genomic features

All genomic data were extracted and processed using UCSC genome browser (http://genome.ucsc.edu/) and Galaxy web service (http://main.g2.bx.psu.edu/) based on hg18 human genome assembly. H3K4Me3 (ENCODE track) and Pol II binding (Yale TFBS track) were used to characterize the promoters of TPGs in available cell lines, extracting −3 kb/+3 kb promoter regions of genes according to the annotated transcription start site (TSS) in RefSeq. Polymerase II (Pol2) peak frequency was computed as the proportion of cell lines having a Pol2 peak in the promoter region. To perform an unbiased comparison, Pol2 peak frequency of all genes was normalized to zero mean and unit standard deviation, separately for hematopoietic and non-hematopoietic cell lines. Lengths of 3′UTRs were obtained from RefSeq and conserved elements were obtained from phastConsElements44way. Predicted microRNA target sites were queried using MirDIP web service (http://ophid.utoronto.ca/mirDIP/) with ‘balanced precision’ option. Alternatively, phastConsElements44wayPrimates track and ‘4 of 12 databases’ query option were used for 3′-UTR analysis and yielded similar results (data not shown).

### Analysis of protein domains

For analysis of putative functions of fusion proteins, retained InterPro domains and protein interaction interfaces were extracted from Ensembl Database (http://www.ensembl.org) via extensive usage of Ensembl Perl API, and from structurally resolved human interactome data [55] based on their positions in relation to the breakpoints (according to TICdb). Briefly, we collected InterPro and protein interaction interface (PII) entries that were located entirely upstream or downstream the position of breakpoint in 5′ or 3′ TPGs, respectively. The list of interaction partners for a given TPG is comprised of all proteins that interact with those PIIs present in the corresponding part of the fusion protein. Annotation and gene ontology (GO) terms (if available) were extracted from InterPro using BioMart and from data provided in [56], respectively. Domains were then manually classified into five broad functional categories based on available annotations: K (kinase), H (histone modification), D (DNA binding), P (protein interaction) or O (other/none). A complete list of protein domains classified according to this criterion is provided as supplementary Table S1. As some InterPro domain features are small and are present in multiple copies in some proteins, this could cause biases when trying to analyze domain composition of fusion proteins. Therefore, instead of raw domain counts we used functional profiles, defined as binary strings indicating if a TPG has any domain of a given function. For domain co-occurrence analysis TPG pairs with unique genes and functional profiles were used.

### Spatial proximity analysis

For analysis of relative position of TPGs in the nucleus we used normalized contact frequency (Hi-C) data from [16], [17]. Clusterization of Hi-C distance data was performed as described in [17], to obtain central and peripheral clusters. Additionally, we also analyzed the three clusters (central, centromere-proximal and centromere-distal) from the original paper. Replication timing (RT) data for the same cell line (GM06990) and several additional samples were downloaded from Replication Domain web service (http://www.replicationdomain.com/) and smoothed using LOESS. Normalized RT values for all RefSeq genes range from −1.5 to 1.5 and we have considered genes with RT>0.5 as early-replicating. Chromosomal positions of central and peripheral, early and late, and TPG-containing loci are presented in supplementary figures S5 and S6. For analysis of lineage-specific differences in TPG distances we used Hi-C data for GM06990 and K562 cell lines [16], [17].

Mitelman database (http://cgap.nci.nih.gov/Chromosomes/Mitelman) was used to provide estimates for clinical frequency of translocations. For this we counted the number of times that each translocation is reported in the database associated to a hematological malignancy. Data were not subject to any regression model, but to simple statistical testing: translocations were categorized as rare (reported only once) and frequent (reported more than once).

All permutation tests, Hi-C distance data clusterization and binomial tests were performed in Matlab. All other statistical tests were performed in GraphPad Prism. As all analyses were performed for large and heterogeneous sets of genes, non-parametric tests were used in most cases to ensure robust results and conclusions. Weka machine learning package (http://www.cs.waikato.ac.nz/ml/weka/) was used to cluster translocations according to their functional profiles. All networks were visualized using Cytoscape software (http://www.cytoscape.org/).

## Note added in proof

A paper by Engretiz et al [57] was published after acceptance of this work, reporting a similar analysis to the one we have performed here. It arrives at quite different conclusions with regard to the importance of nuclear distance in establishing specific combinations of translocation partners. The new report used chromosomal bands involved in translocations from various types of cancer, whereas we focused on the actual genes involved specifically in hematological translocations. As the data and analysis methods are distinct, future studies may reconcile the two findings.

## Supporting Information

Figure S1 Expression of 5′ and 3′ TPGs in dataset #2 (human atlas). **A:** Average expression of 5′ and 3′ TPGs compared to expression of hematological system – related (H/S genes, according to UniProt expression category in DAVID) and all RefSeq genes in hematological tissues (bone marrow, spleen, lymph node and tonsil). **B:** Expression of TPGs in hematopoietic (HEM) tissues compared to non-hematopoietic tissues of epithelial (EPI) and mesenchymal (MES) origin. ***, ns: P<0.001, non-significant, Mann-Whitney test. ###, #: P<0.001, 0.05, Wilcoxon signed rank test.
(TIFF)

Figure S2 5′ TPGs of type 1 (containing domains known to be involved in DNA-binding, protein-interaction, kinase or histone modification) play a role in boosting transcription. Comparison of 5′ TPGs belonging to type 1 (grey boxes) and type 2 (not carrying such domains, white boxes) classes, based on possible expression increase computed as (expression of 5′ TPG/expression of 3′TPG) – 1. Average expression in hematological tissues from datasets #1 (A) and #2 (B). **, *: P<0.01, 0.05; two-tailed T-test.
(TIFF)

Figure S3 Network of 5′TPG interactors, according to protein interaction interfaces retained by 5′ TPGs. Genes that are on average more expressed in hematopoietic samples, than in non-hematopoietic are marked in red.
(TIFF)

Figure S4 Expression (according to dataset#2) of interactors of 3′TPGs. If multiple 3′TPGs have the same interactor, it is counted the corresponding number of times. ### - p<0.001, Wilcoxon matched pairs test.
(TIFF)

Figure S5 Clusterization of corrected Hi-C data into two clusters using k-means algorithm. **A, B**: heatmaps of whole-genome log_2_ Hi-C distances before and after clusterization, respectively (distance is shown as contact frequency, higher values represent shorter distance). Within- and between-cluster distances for resulting clusters are shown with white labels. Larger and smaller clusters clearly represent peripheral and central regions, respectively. **C:** Central (proximal) cluster enrichment trends are same for 2- and 3-cluster clusterization. Odds ratio of frequency in proximal cluster compared to all RefSeq genes and p-values computed based on binomial distribution provided. **D:** Chromosomal locations of TPGs (red) and regions belonging to central cluster (green). **E:** Mean Hi-C reads count measuring contact frequency of TPG-containing loci with all genomic loci on other chromosomes. High contact frequency indicates closeness to center of nucleus. ***, ns - p<0.001, non-significant, Mann-Whitney test. ### - p<0.001, Wilcoxon matched pairs test.
(TIFF)

Figure S6 Analysis of replication time (RT) data. **A:** Clusterization of smoothed RT data into early and late regions. **B**: Early regions (green) and chromosomal locations of TPGs (red) on human karyotype. **C**: RT trends for TPGs in various samples from ReplicationDomain.org. Data for cell lines (GM06990, REH), patient samples (CD4+ T-cells and two AML samples) and cells of non-haematological origin (IMR90, myoblasts) were used. OR – odds ratio of frequency in early regions as compared to all RefSeq genes, P – p-values computed based on binomial distribution. **D**: Raw RT values of TPG-containing loci and all RefSeq genes. *** - p<0.001, non-significant, Mann-Whitney test. ns - non-significant, Wilcoxon matched pairs test.
(TIFF)

Figure S7 Clinical frequency of translocations is not dependent on spatial proximity (A) or replication timing (B) of 5′ or 3′ TPGs.
(TIFF)

Figure S8 Arguments suggesting that nuclear distance does not directly determine the probability of two specific *loci* being fused together in a translocation. **A:** Hi-C distance (expressed as log2 normalized number of contacts, from [22001755]) between selected chromosome pairs. Higher values indicate closer distance. Reported translocations and number of reports (from Mitelman database) displayed inside rectangles. *: P<0.0001, Mann-Whitney U-test. **B:** Hi-C distance between chromosome pairs known (>1) and not reported (0) to be translocated (according to TICdb and Mitelman database). ns: non-significant, Mann-Whitney U-test. **C:** Apparent nuclear closeness of TPGs involved in translocations might result from the association between high-order genomic organization and functional features such as gene activity and replication timing.
(TIFF)

Table S1 List of annotated domains showing InterPro identifiers and their classification as type 1 or type2 domains (see Methods).
(XLSX)
