# Supplementary figures and images for: Genomic Hallmarks of Genes Involved in Chromosomal Translocations in Hematological Cancer
Source: PLoS Comput Biol. 2012 Dec 6;8(12):e1002797. doi: 10.1371/journal.pcbi.1002797 (PMC3516532; doi:10.1371/journal.pcbi.1002797)

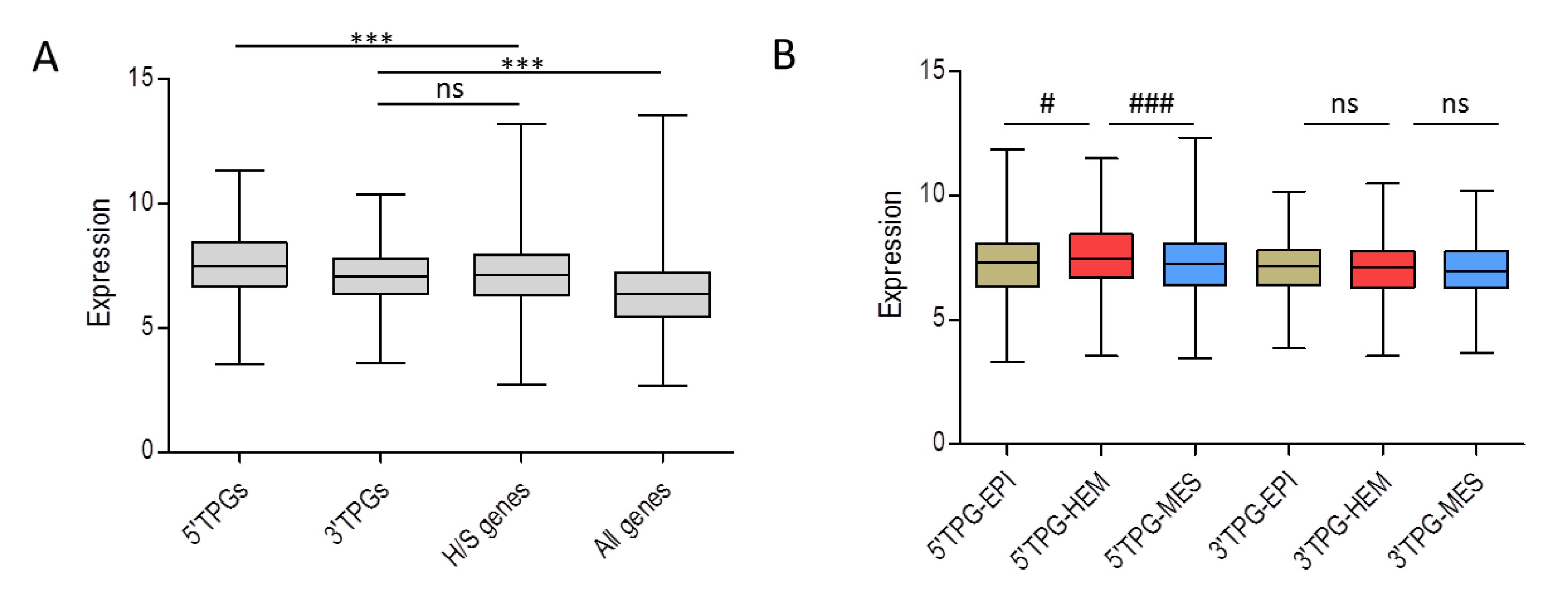

Supplement: Figure S1 — Expression of 5′ and 3′ TPGs in dataset #2 (human atlas). A: Average expression of 5′ and 3′ TPGs compared to expression of hematological system – related (H/S genes, according to UniProt expression category in DAVID) and all RefSeq genes in hematological tissues (bone marrow, spleen, lymph node and tonsil). B: Expression of TPGs in hematopoietic (HEM) tissues compared to non-hematopoietic tissues of epithelial (EPI) and mesenchymal (MES) origin. ***, ns: P<0.001, non-significant, Mann-Whitney test. ###, #: P<0.001, 0.05, Wilcoxon signed rank test. (TIFF) [file pcbi.1002797.s001.tiff]

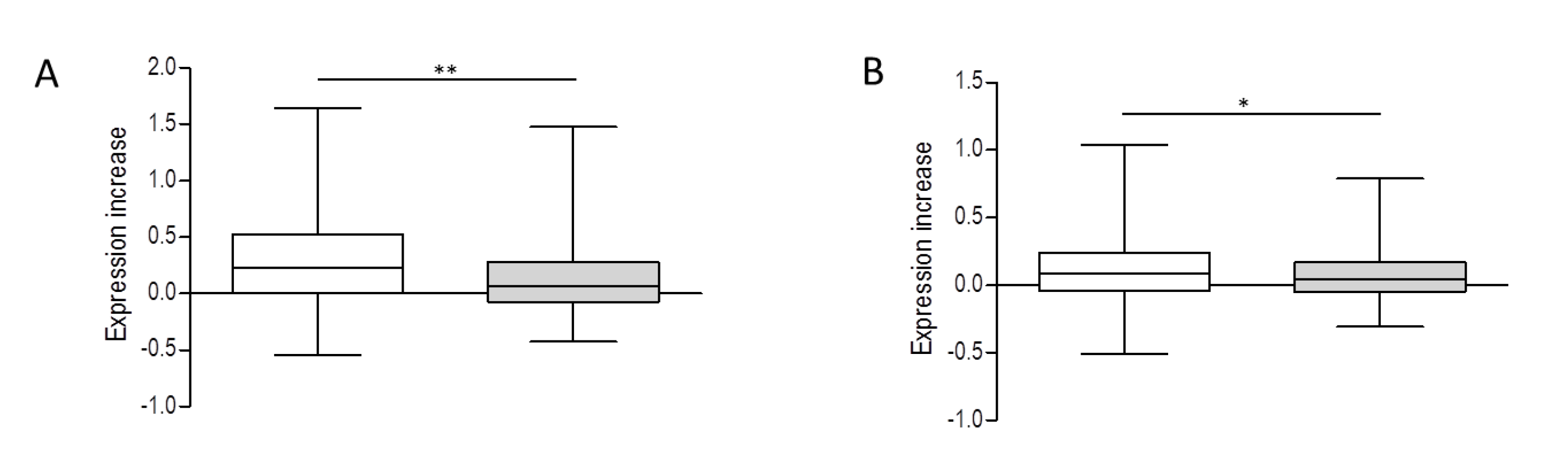

Supplement: Figure S2 — 5′ TPGs of type 1 (containing domains known to be involved in DNA-binding, protein-interaction, kinase or histone modification) play a role in boosting transcription. Comparison of 5′ TPGs belonging to type 1 (grey boxes) and type 2 (not carrying such domains, white boxes) classes, based on possible expression increase computed as (expression of 5′ TPG/expression of 3′TPG) – 1. Average expression in hematological tissues from datasets #1 (A) and #2 (B). **, *: P<0.01, 0.05; two-tailed T-test. (TIFF) [file pcbi.1002797.s002.tiff]

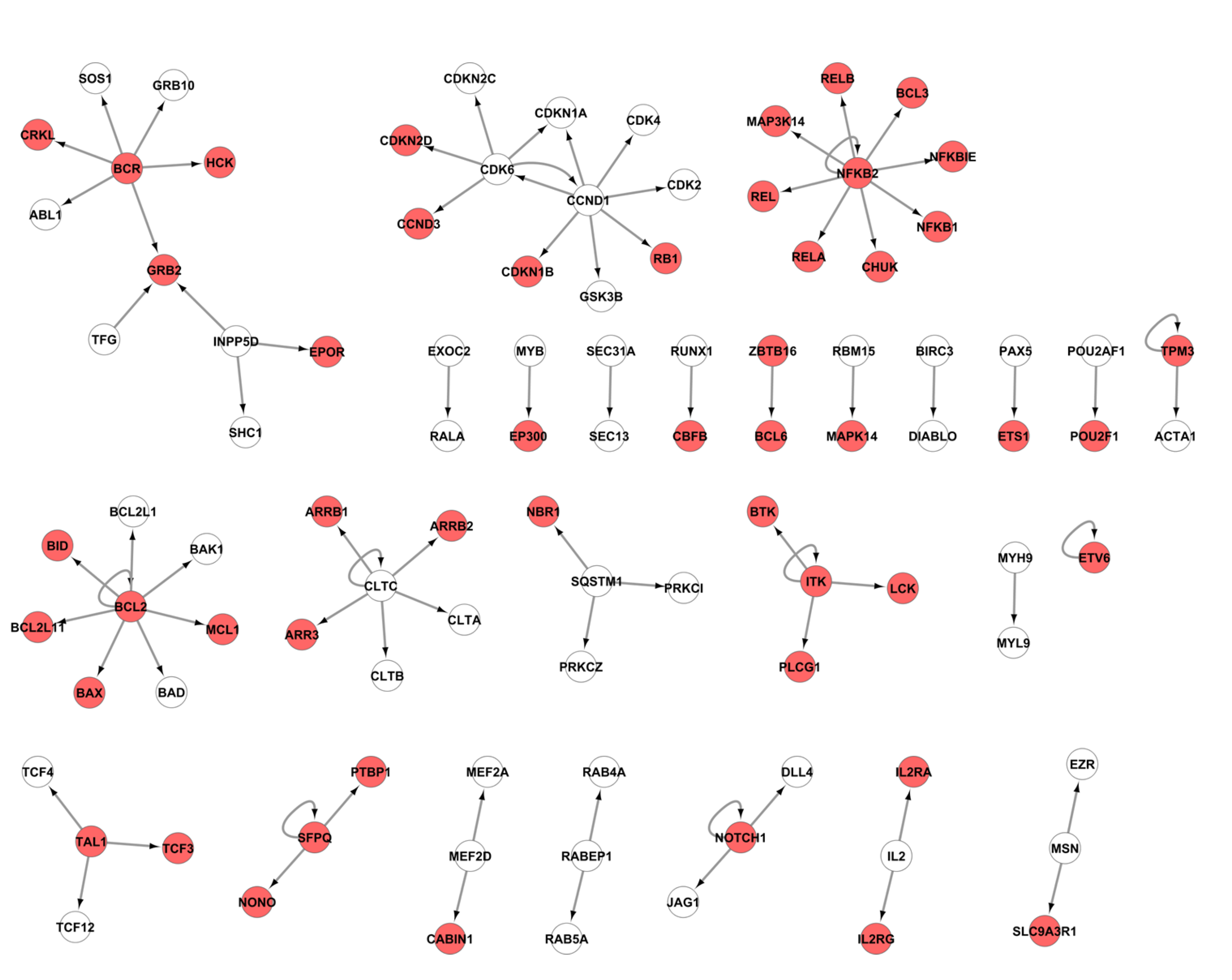

Supplement: Figure S3 — Network of 5′TPG interactors, according to protein interaction interfaces retained by 5′ TPGs. Genes that are on average more expressed in hematopoietic samples, than in non-hematopoietic are marked in red. (TIFF) [file pcbi.1002797.s003.tiff]

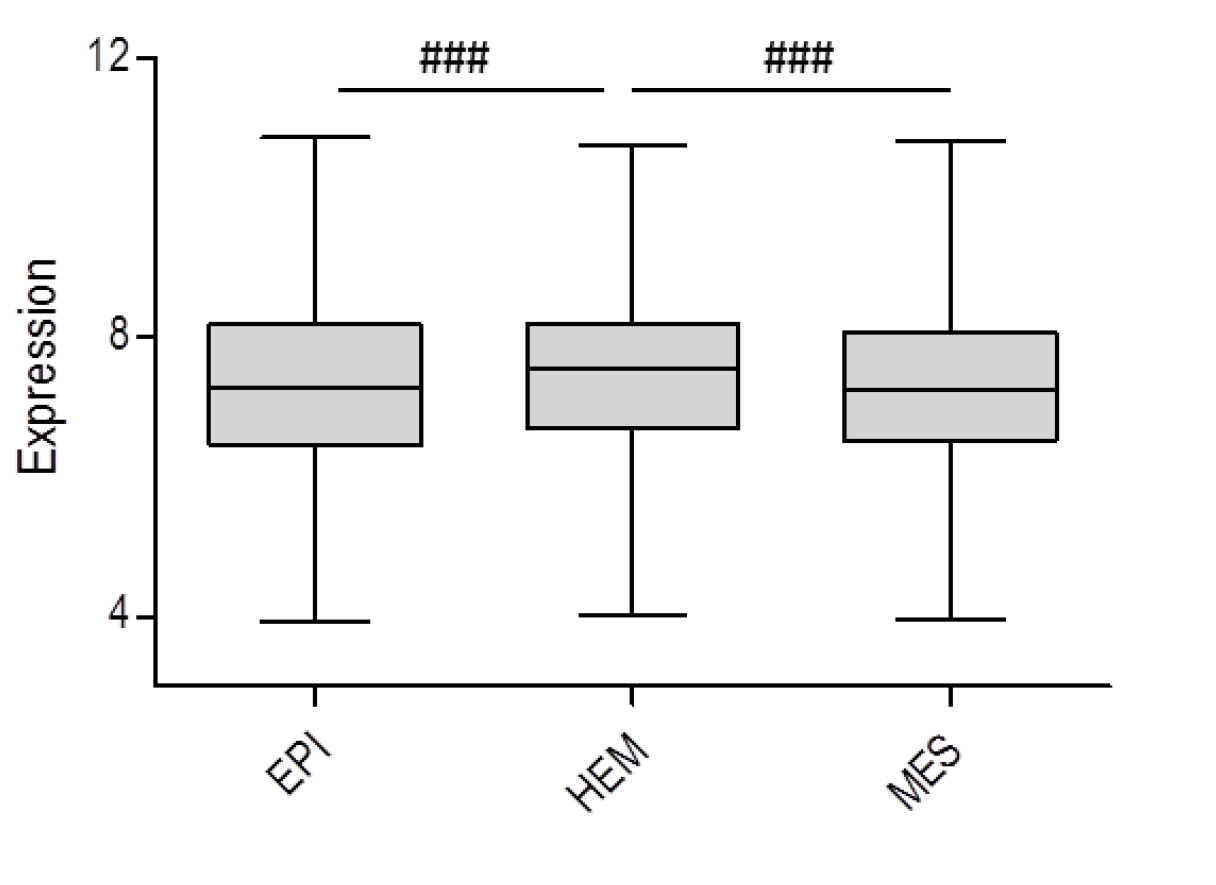

Supplement: Figure S4 — Expression (according to dataset#2) of interactors of 3′TPGs. If multiple 3′TPGs have the same interactor, it is counted the corresponding number of times. ### - p<0.001, Wilcoxon matched pairs test. (TIFF) [file pcbi.1002797.s004.tiff]

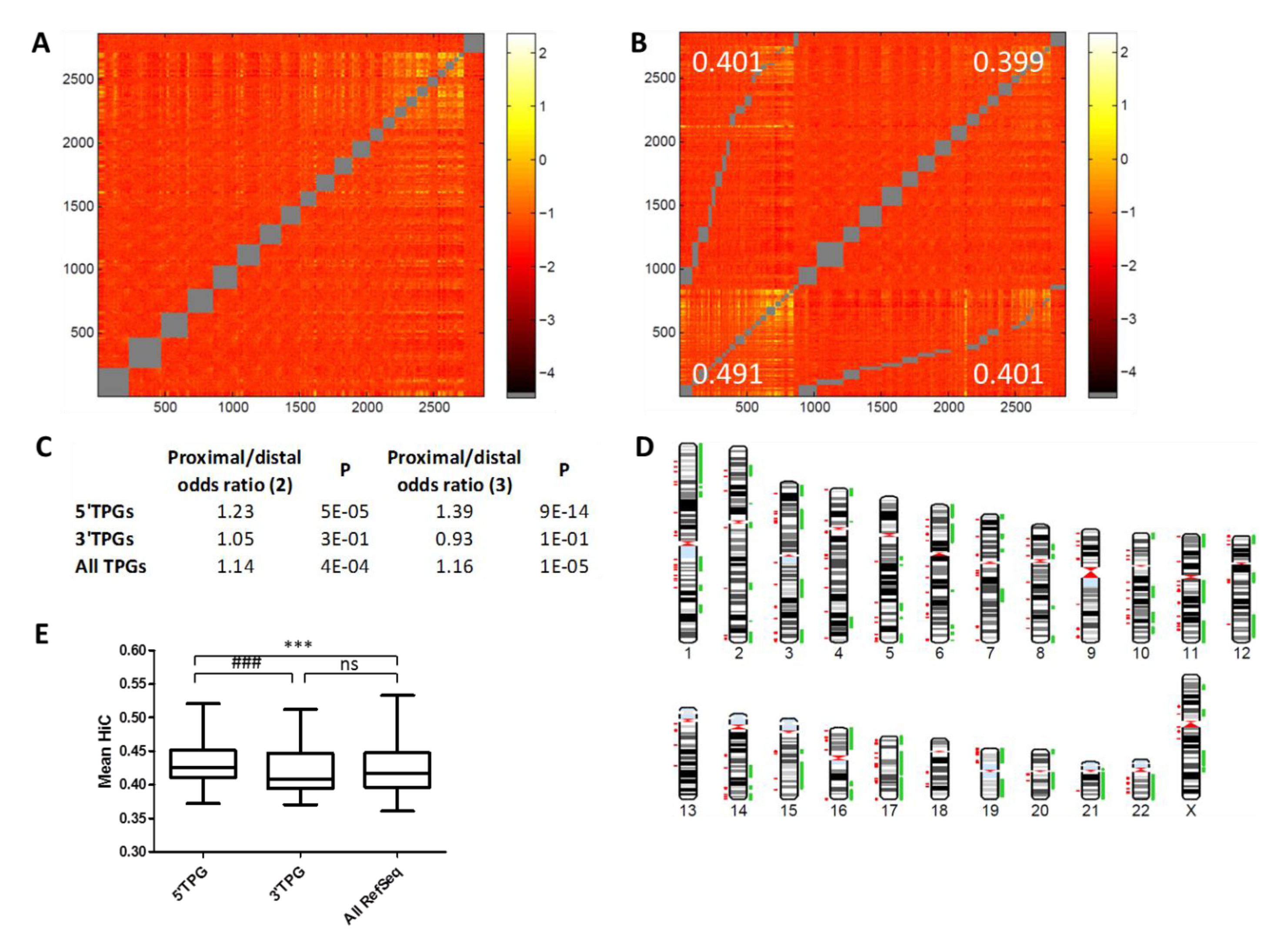

Supplement: Figure S5 — Clusterization of corrected Hi-C data into two clusters using k-means algorithm. A, B: heatmaps of whole-genome log2 Hi-C distances before and after clusterization, respectively (distance is shown as contact frequency, higher values represent shorter distance). Within- and between-cluster distances for resulting clusters are shown with white labels. Larger and smaller clusters clearly represent peripheral and central regions, respectively. C: Central (proximal) cluster enrichment trends are same for 2- and 3-cluster clusterization. Odds ratio of frequency in proximal cluster compared to all RefSeq genes and p-values computed based on binomial distribution provided. D: Chromosomal locations of TPGs (red) and regions belonging to central cluster (green). E: Mean Hi-C reads count measuring contact frequency of TPG-containing loci with all genomic loci on other chromosomes. High contact frequency indicates closeness to center of nucleus. ***, ns - p<0.001, non-significant, Mann-Whitney test. ### - p<0.001, Wilcoxon matched pairs test. (TIFF) [file pcbi.1002797.s005.tiff]

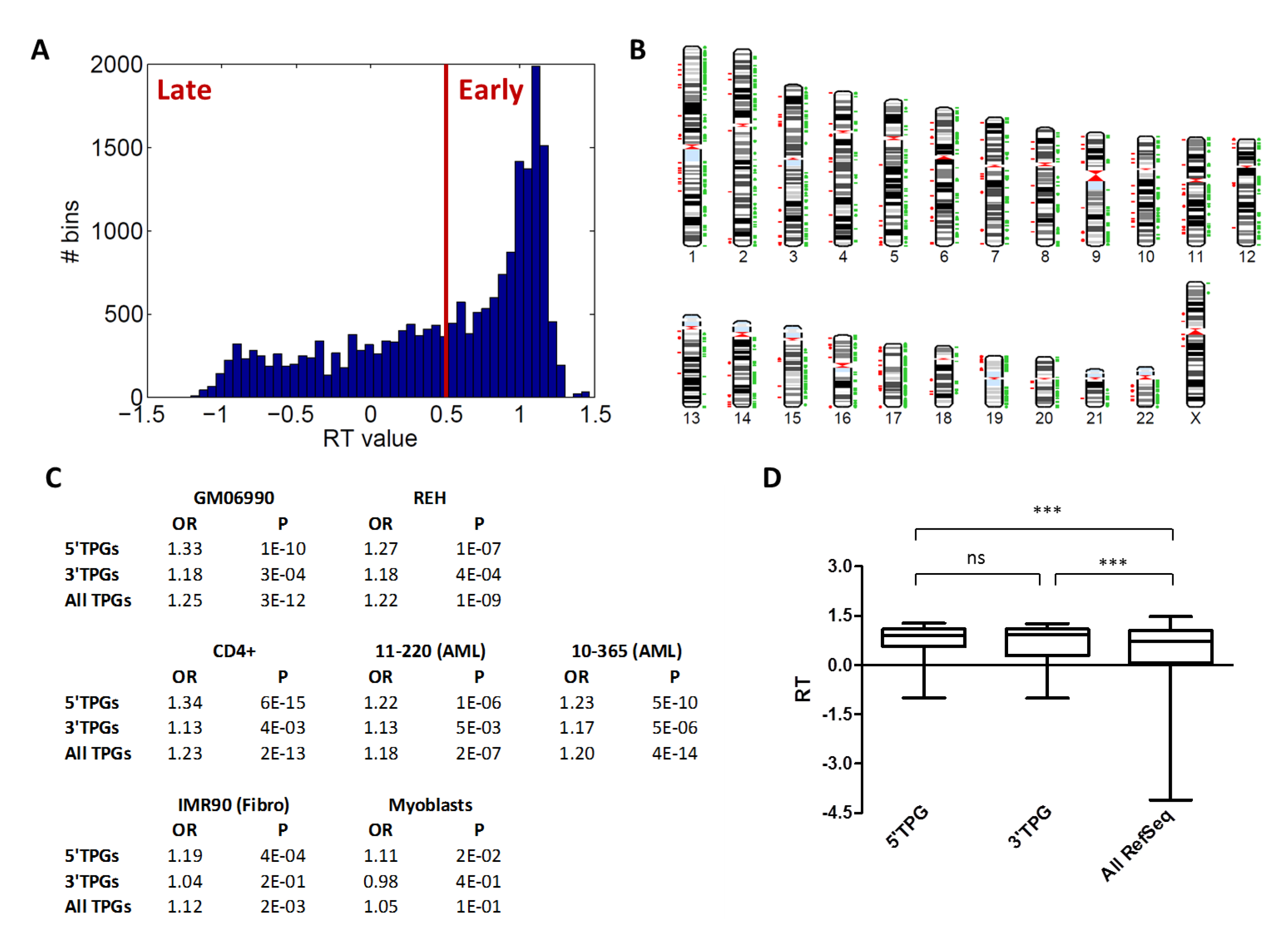

Supplement: Figure S6 — Analysis of replication time (RT) data. A: Clusterization of smoothed RT data into early and late regions. B: Early regions (green) and chromosomal locations of TPGs (red) on human karyotype. C: RT trends for TPGs in various samples from ReplicationDomain.org. Data for cell lines (GM06990, REH), patient samples (CD4+ T-cells and two AML samples) and cells of non-haematological origin (IMR90, myoblasts) were used. OR – odds ratio of frequency in early regions as compared to all RefSeq genes, P – p-values computed based on binomial distribution. D: Raw RT values of TPG-containing loci and all RefSeq genes. *** - p<0.001, non-significant, Mann-Whitney test. ns - non-significant, Wilcoxon matched pairs test. (TIFF) [file pcbi.1002797.s006.tiff]

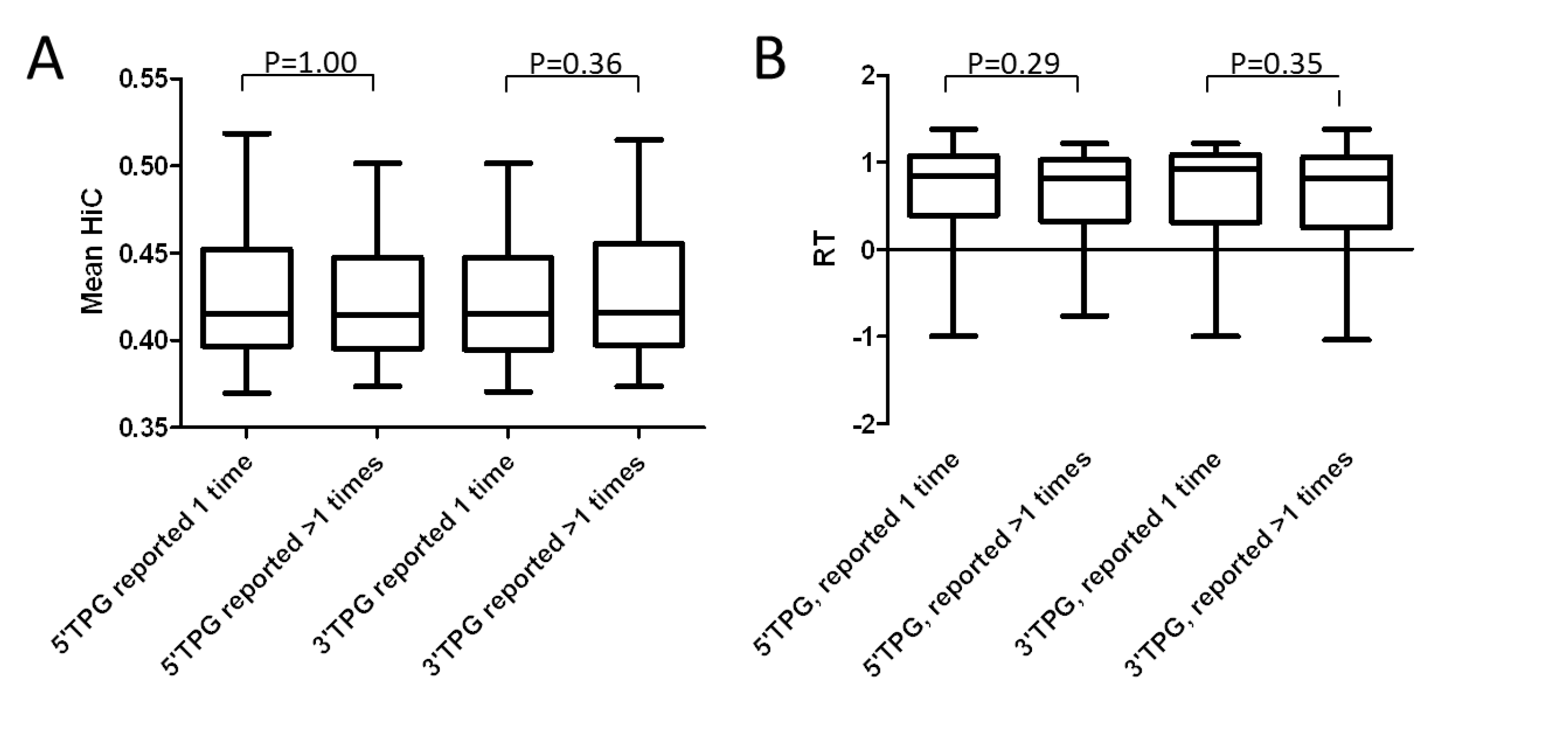

Supplement: Figure S7 — Clinical frequency of translocations is not dependent on spatial proximity (A) or replication timing (B) of 5′ or 3′ TPGs. (TIFF) [file pcbi.1002797.s007.tiff]

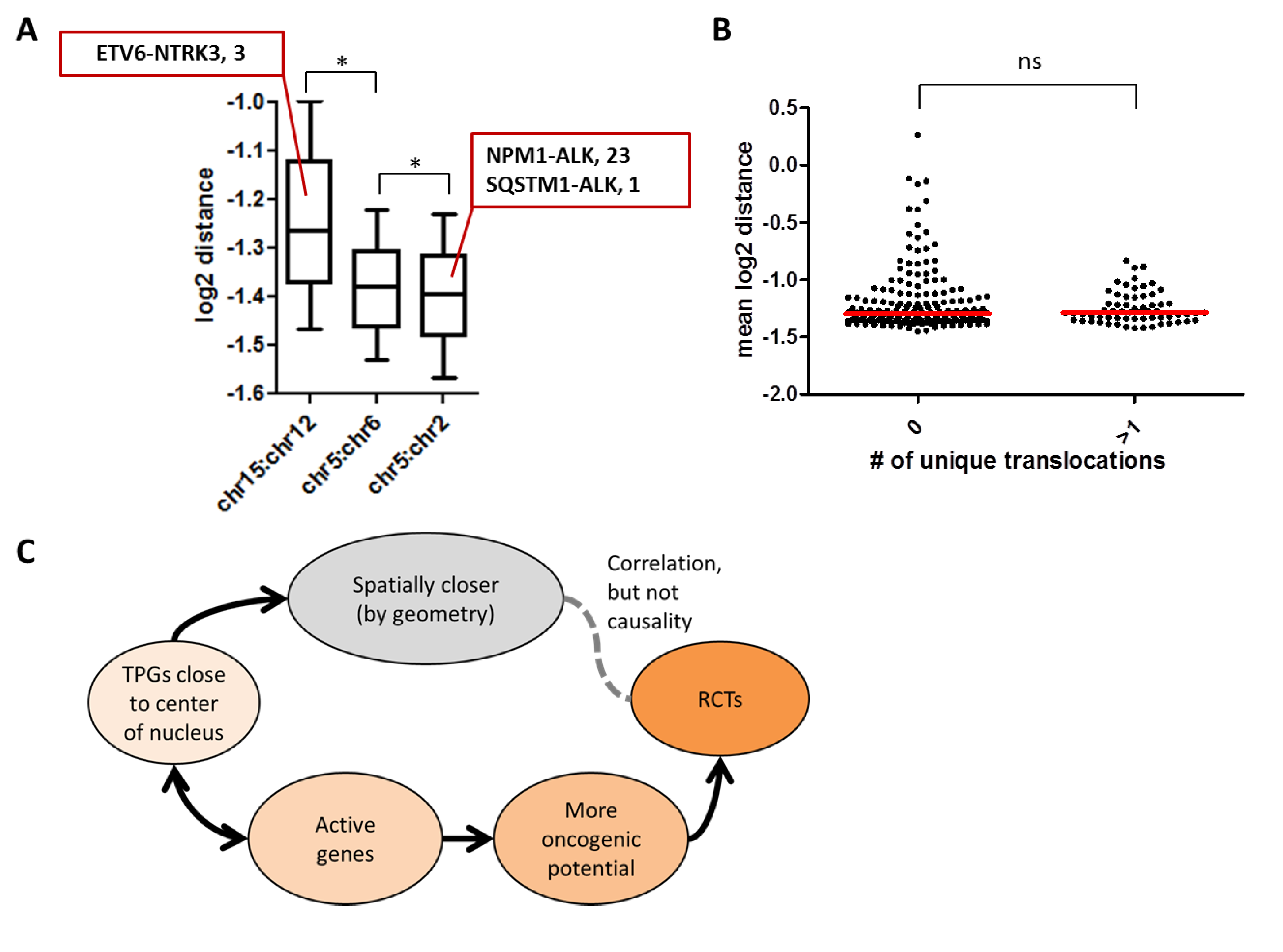

Supplement: Figure S8 — Arguments suggesting that nuclear distance does not directly determine the probability of two specific loci being fused together in a translocation. A: Hi-C distance (expressed as log2 normalized number of contacts, from [22001755]) between selected chromosome pairs. Higher values indicate closer distance. Reported translocations and number of reports (from Mitelman database) displayed inside rectangles. *: P<0.0001, Mann-Whitney U-test. B: Hi-C distance between chromosome pairs known (>1) and not reported (0) to be translocated (according to TICdb and Mitelman database). ns: non-significant, Mann-Whitney U-test. C: Apparent nuclear closeness of TPGs involved in translocations might result from the association between high-order genomic organization and functional features such as gene activity and replication timing. (TIFF) [file pcbi.1002797.s008.tiff]
